# Emotion dataset from Indonesian public opinion

**DOI:** 10.1016/j.dib.2022.108465

**Published:** 2022-07-12

**Authors:** Riccosan, Karen Etania Saputra, Galih Dea Pratama, Andry Chowanda

**Affiliations:** a Computer Science Department, Binus Graduate Program – Master of Computer Science, Bina Nusantara University, Jakarta 11480, Indonesia; b Computer Science Department, School of Computer Science, Bina Nusantara University, Jakarta 11480, Indonesia

**Keywords:** Emotion classification, Dataset, Tweet, Indonesia

## Abstract

An opinion is a type of judgment or a person's point of view about something. Twitter is a popular social media platform that includes a lot of public opinions and would be a suitable location to mine data in text form. With its vast population and active Twitter user base, Indonesia has the potential to be a source of opinion data mining. An opinion may be processed and result in the form of a person's emotional response towards something, such as whether they like, hate, love, or are happy about it. Upon that basis, a dataset of Indonesian-language tweets conveying public opinion on various topics was formed. The fact that there are only limited publicly available emotions text datasets in the Indonesian language supports our basis in this research to form our emotion dataset. The gathered data was cleaned and normalized in the pre-processing stage to the necessary form for study on the task of classifying emotions in Indonesian. The data collected is annotated with six emotional labels: anger, fear, joy, love, sad, and neutral.

**Specifications Table**

**Table:** 

Subject	Data Science, Machine Learning
Specific subject area	This dataset was formed to assist the emotion classification task as part of the sentiment analysis task in Natural Language Processing in Indonesia. This dataset comprises tweets on public opinion in Indonesia on various topics. This data is single-labelled, with six emotional labels: anger, fear, joy, love, sad, and neutral.
Type of data	Text, Table
How the data were acquired	Data was collected using the Twitter API for Developers and the Tweepy Package for Python programming within Google Colab. To acquire data for specific tweets in Indonesia, the search attribute is utilized in the form of lang = 'Id' from Tweepy Package. The data is collected using a dictionary of terms that indicate emotional labels, including the "-filter: retweets" to filter for non-retweet data retrieval*.*
Data format	Raw, Filtered, Annotated
Description of data collection	The data formed contains Indonesian-language tweets. For each emotion label, the data is separated into a “.tsv” file format. The total amount of net data created in the form of tweets and labels is 7080.
Data source location	Indonesia
Data accessibility	The dataset described inside this article and the raw data can be accessed via GitHub using the access link provided. Repository name: GitHub. Direct URL to data: https://github.com/Ricco48/Emotion-Dataset-from-Indonesian-Public-Opinion

**Value of the Data**

• It contains the emotional value of public opinion tweets acquired in Indonesia to carry out sentiment analysis research with the basic task of classifying emotions.
• This data aims to support research in general for sentiment analysis, especially in terms of emotions classification in the Indonesian language.
• This data can be utilized as the primary source or support data for emotion classification research in the Indonesian language or in conjunction with other languages.
• There are only a limited number of datasets available to model emotions classification in the Indonesian language.

## 1 Data Description

**Fig. 1:**
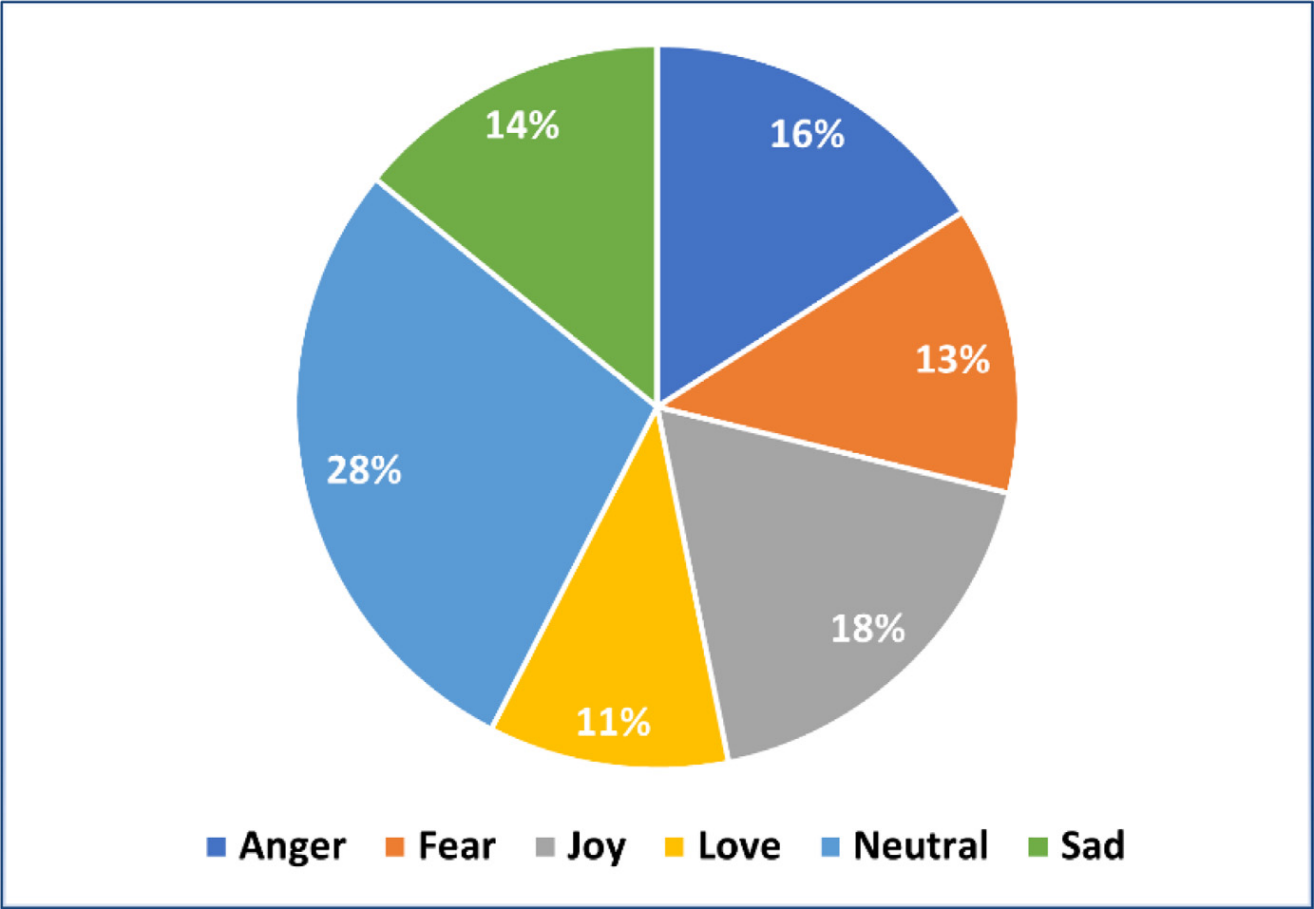
Data distribution percentage base on emotion label.

The emotion dataset created in this study for classifying emotions in the form of Indonesian text was entirely self-gathered using the technique outlined in the following chapter. This dataset was collected from Indonesian tweets containing emotion values from public opinion on various topics in Indonesia. The data was annotated with six emotion labels, namely anger, fear, joy, love, sad, and neutral, with the total amount of data that has been cleaned and fully annotated in the collected dataset being 7,080. Each label has a varied amount of data distribution, as shown in Table 1, including 1130 data for anger, 911 data for fear, 1275 data for joy, 760 data for love, 1003 data for sad, and 2001 data for neutral. Fig. 1. shows the percentage level of data distribution in the collected dataset.

**Table 1:** Data distribution after annotation for final result.

Emotion Label	Anger	Fear	Joy	Love	Sadness	Neutral
Total Data	1,130	911	1,275	760	1,003	2,001

## 2 Methodology & Experimental Design

**Fig. 2:**
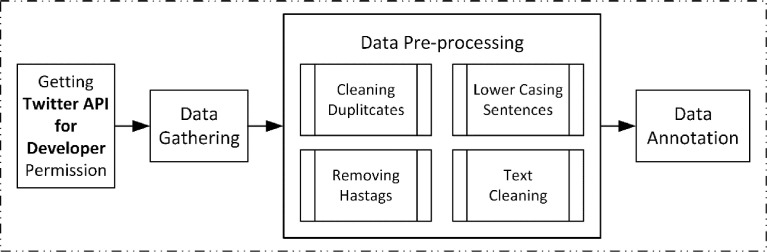
Dataset creation flow.

This research method is illustrated in Fig. 2. The data was collected using the Twitter API for Developers code for accessing the tweet data and the Tweepy package for the data gathering process in Google Colab using the Python programming language. In the Data Gathering Stage, the search property utilizes lang = "id" to get Indonesian tweet data, and a dictionary of words that represent each emotion label, as shown in Table 2 is used for search constraints so that the search for the data per label is more accurate. The data search dictionary's terms and categories are based on the Emotion Hierarchy Level theory [1] as shown in Table 3, which describes the emotional values employed in this study. Because the Joy label emotion has many characteristics with the basic emotion of Happiness described in the article, the Joy label search words and categories adopt the basic form of Happiness's basic emotion hierarchy. An extra attribute termed "-filter: retweet" is used in the Data Gathering stage to avoid obtaining retweets data and therefore decrease data duplication.

**Table 2:** Emotion search words and categories.

Emotion	Search Words (Indonesian) & Categories (*)
Sadness	kecewa, sedih, tangis, sesal, kusut, murung, muram, lesuh, sunyi, sepi, kelam, duka, kemalangan, derita, melankolis, kalah, penolakan, ditolak, simpati, kasihan, abai, kesendirian, dipermalukan
Anger	marah,benci, emosi, jengkel, kesal, dendam, ngamuk, iritasi, terganggu, tidak suka, iri, dengki, siksa, jijik, gerutu, kepahitan, *bentuk umpatan, *kata kasar
Love	suka, cinta, sayang, perhatian, tertarik, nyaman, asmara, kasih, puja, rindu, kangen, lembut, halus, *gairah, *afeksi
Joy	senang, bahagia, puas, membara, tawa, semangat, gembira, ceria, riang, cerah, enak, makmur, terberkati, euforia, bangga, optimis
Fear	takut, ragu, kaget, kejut, palsu, tekanan, histeris, teror, panik, cemas, ngeri, cacat, meleset, gugup, tegang, khawatir, malu, gelisah, *inkonsistensi
Neutral	biasa, normal, santai, pesan, kabar, *berita, *pertanyaan

**Table 3:** Emotion hierarchy in english [1].

Emotion	Subordinate
Love	(1) adoration, affection, love, fondness, liking, attraction, caring, tenderness, compassion, sentimentality; (2) arousal, desire, lust, passion, infatuation; (3) longing
Happiness	(1) amusement, bliss, cheerfulness, gaiety, glee, jolliness, joviality, joy, delight, enjoyment, gladness, happiness, jubilation, elation, satisfaction, ecstacy, euphoria; (2) enthusiasm, zeal, zest, excitement, thrill, exhilaration; (3) contentment, pleasure, pride, triumph; (4) eagerness, hope, optimism; (5) enthrallment, rapture; (6) relief
Anger	(1) aggravation, irritation, agitation, annoyance, grouchiness, grumpiness; (2) exasperation, frustration; (3) anger, rage, outrage, fury, wrath, hostility, ferocity, bitterness, hate, loathing, scorn, spite, vengefulness, dislike, resentment; (4) disgust, revulsion, contempt; (5) envy, jealousy; (6) torment
Fear	(1) alarm, shock, fear, fright, horror, terror, panic, hysteria, mortification; (2) anxiety, nervousness, tenseness, uneasiness, apprehension, worry, distress, dread
Sadness	(1) agony, suffering, hurt, anguish; (2) depression, despair, hopelessness, gloom, glumness, sadness, unhappiness, grief, sorrow, woe, misery, melancholy; (3) dismay, disappointment, displeasure; (4) guilt, shame, regret, remorse, alienation, isolation, neglect, loneliness, rejection, homesickness, defeat, rejection, insecurity, embarrassment, humiliation, insult; (5) pity, sympathy

Next, the acquired data is cleaned up in the Pre-processing stage, with the basic pre-processing step [2] which includes eliminating duplicates, lower-casing tweet sentences, removing hashtags, and lastly, cleaning text from tweet mentions, emoticons, URLs, non-emotion symbols (arrow, underscore, @ sign, per cent and dollar), and excess characters (such as double white space, double coma, etc.). Stop-word normalization was not implemented, and there was no token normalization (one-two character, slang terms, informal words, and short words) in this study to optimize the acquisition of information for the emotion classification in the data.

The Data Annotation Step is the final stage, in which three annotator subjects use six emotional labels, namely anger, fear, joy, love, sad, and neutral, to annotate the data. The annotation in our research employs five basic human emotion categories, namely anger, fear, joy, love, and sorrow, as utilized by Saputri and his team in the Emotion Classification on the Indonesian Twitter dataset [3], which is based on Shaver's theory of basic human emotions [4], which was later popularized by Parrott as Parrott's Basic Emotions [5]. The neutral label was added as the sixth label to accommodate data with characteristics outside of the five basic emotion labels. The use of the six emotion labels in this research is also based on the knowledge gained from the paper A Review on Text-Based Emotion Detection [6], which presents a number of earlier datasets for text-based emotion analysis tasks and the different kinds of emotions contained in them.

We used Kappa's Statistic [7] to compute the match value between the annotators with Cohen's and Fleiss's Kappa after the data was annotated, with the results provided in Table 4. The Cohen's Kappa technique yields a result of 0.5679, whereas the Fleiss's Kappa method yields a score of 0.5657. Based on these two results, the level of agreement for adopting the dataset annotations created by this research is moderate agreement level. The dataset created during the development of the Indonesian text-based emotion classification task was implemented in an experiment after acquiring Kappa's Statistics to serve as a baseline. Multilingual BERT [8], a pre-trained model, is utilized, and resulting in 0.99 and 0.74. for training accuracy and evaluation accuracy, respectively.

**Table 4:** Inter-Annotator Agreement Value with Cohen's and Fleiss's Kappa Method.

Pair	Kappa Value
A1 – A2	0.5747
A1 – A3	0.5713
A2 – A3	0.5578
**Average Cohen's Kappa Value**	**0.5679**
**Fleiss's Kappa Value**	**0.5657**

## Ethics Statements

The relevant informed consent was obtained from the annotator subjects involved in this paper. The data collected to build the dataset from this article has been fully anonymized, and the platform data redistribution policies were complied with. Twitter gives a non-exclusive, royalty-free, non-transferable, non-sublicensable, and revocable license to crawl data for the creation of datasets. Because the data acquired for the research is a non-profit and non-commercial project, it must be identified as non-profit and non-commercial data, according to Twitter's provisions. This dataset may not be used to monitor or way of measuring Twitter API performance.

## CRediT authorship contribution statement

**:** Conceptualization, Writing – original draft. **Karen Etania Saputra:** Data curation. **Galih Dea Pratama:** Data curation. **Andry Chowanda:** Methodology, Validation, Writing – review & editing.

## Declaration of Competing Interest

The authors declare that they have no known competing financial interests or personal relationships that could have appeared to influence the work reported in this paper.

## Data Availability

Emotion Dataset from Indonesian Public Opinion (Original data) (GitHub).
